# Loss of the Tumor Suppressor *Pten* Promotes Proliferation of *Drosophila melanogaster* Cells In Vitro and Gives Rise to Continuous Cell Lines

**DOI:** 10.1371/journal.pone.0031417

**Published:** 2012-02-21

**Authors:** Steven E. Justiniano, Anne Mathew, Sayan Mitra, Sathiya N. Manivannan, Amanda Simcox

**Affiliations:** Department of Molecular Genetics, Ohio State University, Columbus, Ohio, United States of America; University of North Carolina, United States of America

## Abstract

In vivo analysis of *Drosophila melanogaster* has enhanced our understanding of many biological processes, notably the mechanisms of heredity and development. While in vivo analysis of mutants has been a strength of the field, analyzing fly cells in culture is valuable for cell biological, biochemical and whole genome approaches in which large numbers of homogeneous cells are required. An efficient genetic method to derive *Drosophila* cell lines using expression of an oncogenic form of *Ras (Ras^V12^)* has been developed. Mutations in tumor suppressors, which are known to cause cell hyperproliferation in vivo, could provide another method for generating *Drosophila* cell lines. Here we screened *Drosophila* tumor suppressor mutations to test if they promoted cell proliferation in vitro. We generated primary cultures and determined when patches of proliferating cells first emerged. These cells emerged on average at 37 days in wild-type cultures. Using this assay we found that a *Pten* mutation had a strong effect. Patches of proliferating cells appeared on average at 11 days and the cultures became confluent in about 3 weeks, which is similar to the timeframe for cultures expressing *Ras^V12^*. Three *Pten* mutant cell lines were generated and these have now been cultured for between 250 and 630 cell doublings suggesting the life of the mutant cells is likely to be indefinite. We conclude that the use of *Pten* mutants is a powerful means to derive new *Drosophila* cell lines.

## Introduction

The establishment of cell lines from human tissues involves genetic manipulation of telomerase, tumor suppressors and oncogenes. Telomerase is required to circumvent the finite number of divisions most somatic cells experience due to telomere shortening [1]. In human cells, telomerase expression together with mutations in tumor suppressors leads to immortality [2]. Rodent cells, in contrast to human cells become immortal spontaneously at high frequency [3]. Expression of oncogenes such as Ras allows cells to be independent of growth factors [2]. Expression of oncogenic Ras in human primary cells that lack telomerase activity causes senescence, but we discovered expressing a *Ras* oncogene (*Ras^V12^*) in *Drosophila* primary embryonic cells promotes cell proliferation to rapidly give rise to immortal cell lines [4], [5], [6]. This different response may be because *Drosophila* maintains telomere length without telomerase [7]. Expression of *Ras^V12^* has proved to be a useful genetic tool to create *Drosophila* mutant cell lines [4], [8], [9]. By analogy with mammalian cells, inactivation of tumor suppressors could provide another genetic means to immortalize *Drosophila* cells. To test this idea we surveyed a collection of *Drosophila* tumor suppressor mutants for their ability to promote proliferation of cells in culture.

Homologs of many mammalian tumor suppressor genes are conserved in *Drosophila* and new tumor suppressors have been discovered in genetic screens using flies. These include both whole organism screens for larval-pupal lethals with overgrowth phenotypes in the imaginal discs and screens for tumors that develop as clonal patches in adults (reviewed in [10]). Analysis of these genes in *Drosophila* has made important contributions to understanding the biology of tumor suppressors and in a number of cases has supported the involvement of these genes in human cancers (reviewed in [10]).

*Drosophila* tumor suppressors are broadly divided into two classes; neoplastic and hyperplastic that distinguish their different overgrowth phenotypes [10], [11]. The first *Drosophila* neoplastic tumor suppressor isolated, *lethal (2) giant larvae (lgl)* [12], [13], was later found to be part of complex called the Scribble complex (Scib/Dlg/Lgl), which is required for the establishment and maintenance of cell polarity in epithelia (reviewed in [11], [14], [15]). Loss of function mutations in these and other neoplastic tumor suppressor genes leads to an increase in cell number, and a failure to terminally differentiate. The second class, hyperplastic tumor suppressors, determine proper tissue size by regulating the number of cells in an organ or tissue (reviewed in [11]). *Pten* and some members of the Hippo pathway are well-characterized examples of hyperplastic tumor suppressors [11], [16], [17]. Loss of function mutations in hyperplastic tumor suppressors cause an increase in cell number, although the ability of the cells to differentiate is not compromised.

Here we tested mutations in both classes of tumor suppressor genes for their ability to promote proliferation of *Drosophila* cells *in vitro*. We found that a *Pten* mutation had a dramatic effect on primary cultures. The cultures rapidly became confluent and gave rise to continuous lines. This identifies *Pten* mutation as a second genetic approach for generating cell lines in *Drosophila*.

## Results and Discussion

### Primary-culture assay to determine when proliferating cells appear in tumor-suppressor mutant cultures relative to wild type and *Ras^V12^*-expressing cultures

Wild-type embryonic primary cultures follow a pattern of development that initially involves the appearance of morphologically distinct types of terminally differentiated cell types such as muscle, nerve and fat body (reviewed in [18]). Patches of proliferating spindle-shaped, and more rarely epithelial-like, cells emerge much later than these differentiated cells types and are likely to be a major cell type that gives rise to continuous lines [18]. These proliferating cell patches appear in wild type cultures after a delay of several weeks, are often transient, and typically occur in waves over many months with only the later ones giving rise to persistent populations [18].

**Figure 1:**
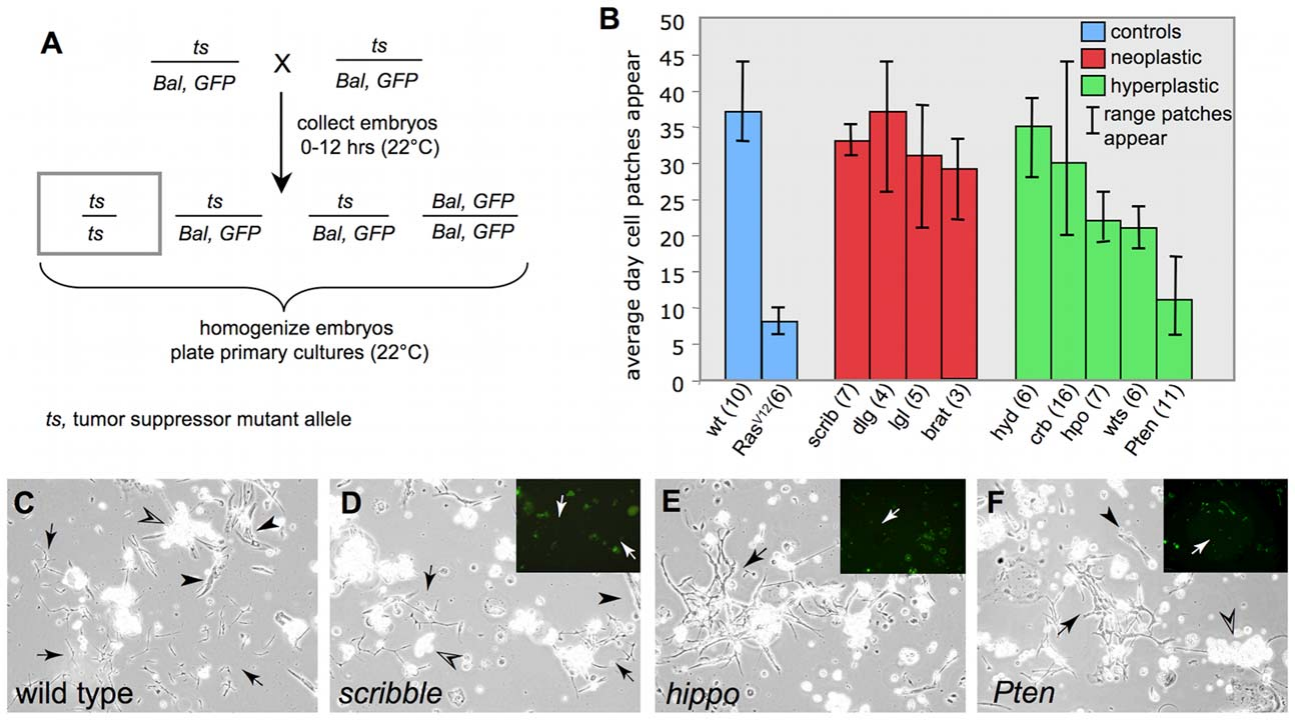
Time of appearance of proliferating cell patches in wild-type and tumor-suppressor mutant primary cultures. (A) Cross to generate embryos for tumor suppressor primary cultures. Tumor suppressor *(ts)* mutants were maintained in stocks with a marked balancer chromosome that expresses a *GFP* transgene *(Bal, GFP)*. One quarter of the progeny embryos are the desired class (boxed genotype). Primary cultures were established from mixed embryos and cells homozygous for the *ts* allele could be recognized because they are GFP negative. (B) The average day, and the range of days at which proliferating cells appear is shown. The number of cultures for each genotype is given (n). Wild-type control and *Act-Gal4; UAS-Ras^V12^* positive control (blue); neoplastic mutants (red); hyperplastic mutants (green). The appearance of proliferating cells in *Pten* and *Ras^V12^* cultures was significantly earlier than in wild-type control cultures (P<0.001). The appearance of proliferating cells in *wts* and *hippo* cultures was also significantly earlier than in wild-type control cultures (P<0.05). There were no significant differences between wild-type control cultures and any of the neoplastic mutants. See also Figure S1. (C–F) Examples of cultures showing proliferating patches that first appeared on average at about 33–37 days in wild type (C) and *scribble* (D), 21 days in *hippo* (E) and 11 days in *Pten* (F). Differentiated cell types such as muscle (arrowhead) and fat (open arrowhead) are present in all genotypes. Insets in D–F show a GFP image demonstrating that the indicated patches of proliferating cells (arrows) were negative for GFP and therefore of the mutant genotype.

In this study, wild-type cultures followed the expected pattern with patches of proliferating cells emerging on average at day 37 with a range spanning a few days in individual cultures (Figure 1B and 1C). These serve as a control to identify genotypes in which these cells appear earlier, such as cultures expressing oncogenic *Ras^V12^ (Act5C-Gal4; UAS- Ras^V12^).* In *Ras^V12^* cultures, patches of proliferating cells appeared on average at about day 8 (Figure 1B). Changes in the timing of appearance and persistence of proliferating cells can be an indicator of genotypes that will readily give rise to continuous cell lines as we discovered for primary cultures expressing *Ras^V12^* that readily progressed to continuous cell lines [4]. Thus, cultures expressing transgenic *Ras^V12^* served as a positive control in these assays.

We used the time of appearance of proliferating cell patches, in comparison with wild-type and *Ras^V12^-*expressing cultures, as an assay for the effects of tumor suppressor mutations to discover which, if any, had positive effects and could provide a means for deriving cell lines. Cultures were established from the progeny of flies heterozygous for loss-of-function tumor suppressor mutations (Table 1; Figure 1A). The cultures were, therefore, derived from embryos of four genotypes but the homozygous mutant cells could be distinguished in the cultures, as all other genotypes expressed the marker gene *Green Fluorescent Protein (GFP)* under the control of the ubiquitous promoters *Actin5C* or *Ubiquitin* (Figure 1A). Several primary cultures were established for each genotype and examined every few days over a period of about 8 weeks. The range of days on which patches of proliferating cells appeared in a given culture was determined and the GFP marker allowed us to infer the genotype of the cells. The results are shown in Figure 1B and discussed below.

**Table 1:** Tumor suppressors tested in in vitro assays.

Gene (allele used*)	Mammalian homolog	Tumor suppressor class and Complex or Pathway	GO classification**	
			Molecular function	Biological Process
*scribble (scrib^1^)*	*Scrib*	neoplastic Scrib/Dlg/Lgl	kinase regulator	signal transduction cell adhesion
*discs large 1 (dlg^XI-2^)*	*Dlg1*	neoplastic Scrib/Dlg/Lgl	unknown-protein interaction domains	signal transduction cell adhesion
*lethal (2) giant larvae (lgl^4^)*	*Llgl1*	neoplastic Scrib/Dlg/Lgl		signal transduction cell adhesion
*brain tumor (brat^11^)*	*TRIM*	neoplastic Myc regulation	E3 ubiquitin-protein ligase	cell cycle nervous system
*hyperplastic discs (hyd^35^)*	*EDD*	hyperplastic	ubiquitin-protein ligase activity	protein metabolic process
*crumbs (crb^2^)*	*Crb1*	hyperplastic wts/hpo pathway	transmembrane EGF	apical/basal polarity
*hippo (hpo^BF33^)*	*MST1/2*	hyperplastic wts/hpo pathway	kinase	signal transduction apoptosis cell cycle
*warts (wts^MGH1^)*	*LATS1/2*	hyperplastic wts/hpo pathway	kinase	signal transduction apoptosis cell cycle
*Pten (Pten^117^)*	*Pten*	hyperplastic insulin pathway	phosphatase	cell cycle, signal transduction

*The allele used in this study is indicated. All alleles used were loss-of-function and when possible null alleles. The alleles and their sources are described in the methods section.

**GO classification adapted from Panther (http://www.pantherdb.org/).

### Neoplastic tumor suppressor mutants have in vitro growth characteristics that are similar to wild type

We tested four tumor suppressor mutants, from the neoplastic class (Table 1). *scribble (scrib), discs large 1 (dlg),* and *lgl,* which are part of the Scribble complex, and *brain tumor (brat)*. The mutants have overgrowth phenotypes in imaginal discs and neural tissues and can form tumors when implanted into host flies [10], [14], [19]. For each mutant strain, multiple primary cultures were established and the time at which patches of proliferating cells emerged was determined (Figure 1B). Cultures of each genotype developed patches of spindle-shaped cells on average between 29 and 37 days (*scrib,* 33; *dlg,* 37; *lgl,* 31; *brat,* 29; Figure 1B–D). The range of days over which these cells appeared overlapped that of wild-type cultures (Figure 1B). In the long term, the cultures grew slowly taking on average between 11 and 13 weeks to reach confluence. In contrast, *Ras^V12^*-expressing cultures, and as we show below *Pten* mutant cultures, reach confluence in about three weeks [4]. We conclude use of the neoplastic mutants, which we tested, is not a practical approach for the development of cell lines.

### Some hyperplastic tumor-suppressor mutants promote cell proliferation in vitro—Pten is the most potent

We tested 5 hyperplastic tumor suppressor mutants including the *hyperplastic discs (hyd)* mutant [20] and 4 genes from two major signaling pathways; the Hippo pathway *(crumbs (crb), hippo (hpo)* and *warts (wts))* (reviewed in [17]) and the insulin pathway *(Pten)* [16], [21], [22].

#### hyd

*hyd* mutants show a variable disc overgrowth phenotype as homozygous mutants, which led to classification of the gene as a tumor suppressor [20]. In the *in vitro* assay, proliferating patches appeared on average at 35 days in *hyd* mutant cultures, which is similar to the time frame for wild type (Figure 1B). In keeping with the idea that *hyd* mutant cells do not have increased ability to proliferate, clonal mutant patches are small and out competed by surrounding wild-type cells and some discs in homozygous mutants are small [20] [23]. The gene has also been found to be overexpressed, rather than lost, in some human tumors [24].

#### Hippo pathway

*crb* mutants represent the group of hyperplastic tumor suppressors that function upstream in the Hippo pathway *(crb, expanded, merlin* and *fat)* [17], [25], [26], [27], [28]. Cultures derived from *crb* mutants develop proliferating patches on average at 30 days (Figure 1B). Primary cultures from core members of the Hippo pathways *(hpo* and *wts)* developed patches of proliferating cells earlier on average at 22 or 21 days, respectively (Figure 1B and 1E). This was significantly shorter than the time of appearance of these cells in wild-type cultures (P<0.005) (Figure 1B; Figure S1). The longer time for the appearance of proliferating patches in *crb* mutant cultures is in keeping with the evidence that genes acting upstream of the core Hippo pathway appear to play modulating roles which may be small and additive [17]. Finding that *wts* mutants lead to the earlier appearance of proliferating cells is in keeping with our finding that reducing *wts* function with RNAi also promoted the development of proliferating cell patches when compared to controls [4]. Thus mutants of core tumor suppressor genes in the Hippo pathway can be used to accelerate the derivation of new cell lines and indeed we have generated cell lines by expressing *wts^RNAi^* [4].

#### Insulin pathway

The occurrence of proliferating cell patches was earliest and most pronounced in cultures derived from mutants of the insulin pathway gene *Pten*, where they appeared in large numbers on average by 11 days (Figure 1B and 1F). This was significantly shorter than the time of appearance of these cells in wild-type cultures (P<0.001) (Figure 1B; Figure S1). We tracked the development of *Pten* mutant primary cultures over longer periods and found they mirrored the development of cultures expressing *Ras^V12^*: they reached confluence at about 3 weeks and the 10^th^ passage by 5 months (Table 2; [4]). The time of the appearance of proliferating cells in *Pten* cultures compared with *Ras^V12^* cultures was not significantly different (Figure S1). Three cell lines were generated (Pten-1, -1A and -X; Table 2). These lines have been passaged multiple times representing between 250 and 630 cell doublings and thus can be considered immortal. The lines were established from the null allele, *Pten^117^,* which has a deletion in the coding sequence that causes a frame shift [29], and the cell line genotype was confirmed using PCR (see methods). Together these results support the use of a loss-of-function *Pten* mutation as a practical strategy for deriving new *Drosophila* cell lines.

**Table 2:** Generation and characteristics of *Pten* cell lines in reference to other cell lines.

Cell line	Weeks to confluence	Months to passage 10	Doubling time (h) (22°C)	Confluent density (×10^6^)*
Pten 1	3	5	43	2.3
Pten 1A	3	5	40	3.2
Pten X	N/D	N/D	42	2.3
Ras 3	3	4	31	7.0
Ras 7	3	4	32	8.9
Pten Ras 8	5	4	36	3.5
Pten Ras 9	5	4	36	3.2
S2	N/A	N/A	33	6.5

*surface area = 3.8 cm^2^.

ANOVA and a Tukey-Kramer multiple comparison test showed that Pten and Pten; Ras lines had a significantly lower confluent density than Ras lines (P<0.01) (F value = 31.052 = MStreatment/MSresidual).

### Pten^117^ mutant and Ras^V12^-expressing primary cells show comparable accelerated growth in culture, but the combination has no added effect

As loss of *Pten* accelerated the appearance of proliferating cells, when compared to wild type and all other tumor suppressor mutants tested (Figure 1B), the mutant primary cultures were examined more closely and compared with the development of *Ras^V12^* expressing cultures. We also tested whether simultaneous loss of *Pten* and expression of *Ras^V12^* had an additive effect. Cultures were established from controls, and stocks that gave rise to *Pten* mutant embryos, embryos expressing transgenic *Ras^V12^*, and *Pten* mutant embryos expressing transgenic *Ras^V12^*.

**Figure 2:**
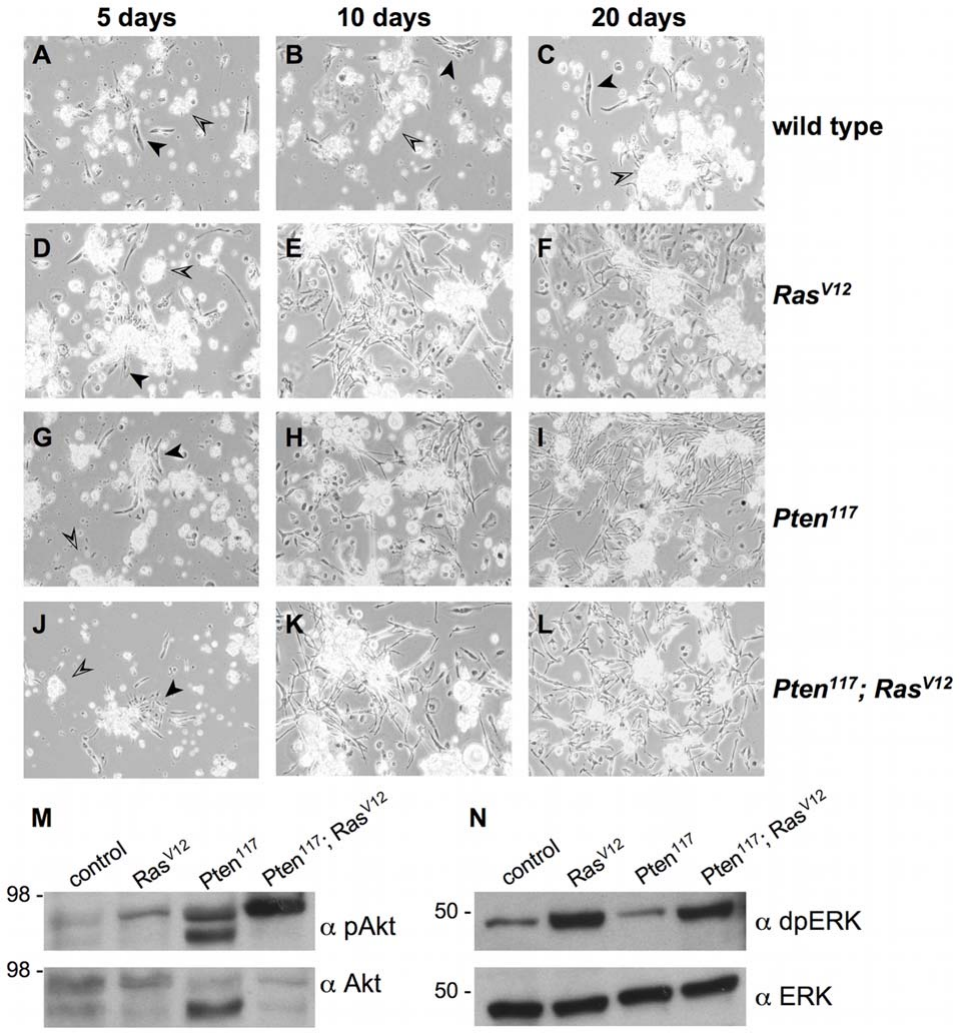
*Pten* mutant primary cultures mirror the development of primary cultures expressing oncogenic *Ras^V12^*. (A–C) Wild type. (D–F) *Ras^V12^ (Act5C-Gal4; UAS-Ras^V12^).* (G–I) *Pten^117^*. (J–L) *Pten^117^; Ras^V12^ (Pten^117^; Act5C-Gal4/Pten^117^; UAS-Ras^V12^).* After five days, primary cultures of all genotypes (A, D, G, J) had differentiated cell types including fat body (open arrowheads) and muscle (arrowheads). After ten days, wild-type cultures (B) had only the same differentiated cell types, whereas, cultures of the other genotypes (E, H, K) had patches of spindle-shaped cells. After 20 days, wild-type cultures (C) had only the same differentiated cell types, whereas, cultures of the other genotypes (F, I, L) were densely populated with spindle-shaped cells. (M and N) Western-blot analysis of primary culture extracts with cells of the indicated genotypes. (M) The Akt pathway is activated (pAkt) above control (wild type) levels in cultures with *Ras^V12^* expressing cells *(Ras^V12^), Pten* mutant cells (*Pten^117^*) and *Pten* mutant cells expressing *Ras^V12^ (Pten^117^; Ras^V12^).* The Erk pathway is activated (dpErk) above control (wild type) levels in cultures with *Ras^V12^* expressing cells *(Ras^V12^)* and *Pten* mutant cells expressing *Ras^V12^ (Pten^117^; Ras^V12^).* Total Akt and Erk, as detected by α-Akt and α-Erk were used as loading controls [40], [41]. Akt is detected as two bands (Cell Signaling Technology) [31]. For unknown reasons the lower band is more prominent in the *Pten* mutant cultures.

Initially, the cell types and their morphologies were very similar in all cultures (Figure 2A, 2D, 2G, 2J), however, after 10 days, there were marked differences between the mutant and control genotypes. *Pten, Ras^V12^*, and *Pten; Ras^V12^* cultures had numerous small patches of spindle-shaped cells and more rarely patches of epithelial-like cells (Figure 2E, 2H, 2K), whereas control cultures had only differentiated cell types (Figure 2B). By 20 days these differences were more pronounced and the mutant cultures were densely populated with proliferating cells (Figure 2F, 2I, 2L). At this stage control cultures still remained sparse and comprised of differentiated cell types (Figure 2C). The *Pten* mutant cultures also expressing *Ras^V12^* developed in a similar fashion to those mutant for *Pten* alone or those just expressing *Ras^V12^*. We generated two cell lines from *Pten; Ras^V12^* cultures and these also developed in a similar fashion to those derived from cells with the single genetic changes (Table 2).

**Figure 3:**
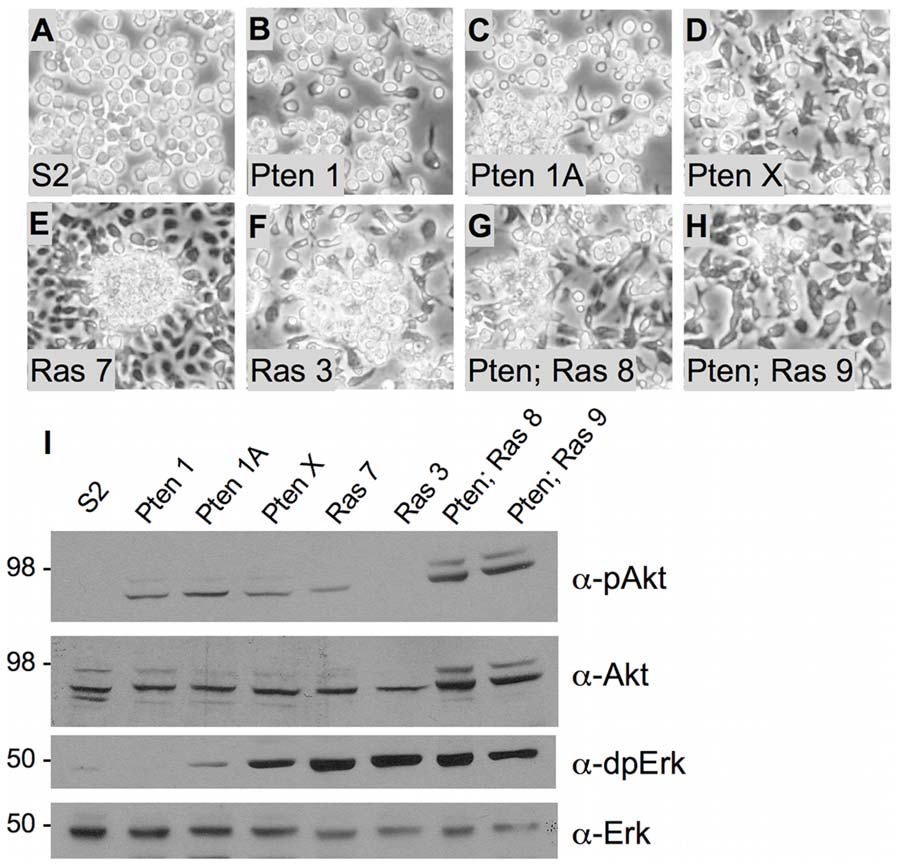
Morphological and molecular comparison of *Pten^117^* cell lines with S2, *Ras^V12^* and *Pten^117^; Ras^V12^* cell lines. (A–H) Cells are shown after 6 days of growth from the same starting cell number. (A) S2. Cells show the typical round morphology and are loosely attached to the surface. (B–D) *Pten^117^*. Pten 1 and 1A have round and spindle-shaped cells. Cells in the Pten 1 line are more loosely attached. Cells in line Pten X are primarily spindle shaped. (E–F) *Ras^V12^*. Ras 7 and 3 have spindle shaped cells that form dense attached clusters. (G–H) *Pten^117^; Ras^V12^.* Pten; Ras 8 and 9 have spindle shaped cells that form small clusters. (I) Western-blot analysis of cell extracts from the indicated lines. The Akt pathway is activated (pAkt) above control levels in all lines with *Pten* mutations. The Ras 7 line also shows elevated activation. The Erk pathway is activated (dpErk) in most lines with very low levels in S2 cells and undetectable levels in line Pten 1. Total Akt and Erk, as detected by α-Akt and α-Erk were used as loading controls [40], [41]. Akt is detected as two bands (Cell Signaling Technology) [31].

We examined pathway activation in the primary cultures and found as expected that Akt signaling was activated in *Pten* mutant cultures (Figure 2M). Pten is a key negative regulator of Akt signaling. We also examined MAPK/Erk signaling as determined by phosphorylation of *Drosophila* Erk a downstream effector of Ras signaling. *Pten* cultures showed wild-type levels of Erk activation (Figure 2N). As expected, primary cultures expressing *Ras^V12^* had high levels of Erk activation (Figure 2N). It is known that *Drosophila* Ras^V12^, but probably not endogenous Ras, can also signal through the Akt pathway [4], [30], [31]. In keeping with our previous findings for Ras^V12^ expression *in vitro,* we also observed enhanced Akt activation in *Ras^V12^*-expressing primary cultures ([4]; Figure 3M). The *Pten*; *Ras^V12^* cultures showed strong activation of both pathways (Figure 2M, 2N). We conclude that the combination of molecular changes resulting from the different genetic backgrounds does not result in additive effects at the cellular level that can further enhance cell proliferation.

### Characteristics of Pten, Ras^V12^, and Pten; Ras^V12^ cell lines

We compared characteristics of *Pten^117^* and *Pten^117^; Ras^V12^* cell lines with *Ras^V12^* lines and cells of the commonly used *Drosophila* S2 line [32]. The cells have different morphologies likely reflecting different cell types (Figure 3A–H). All lines, including S2, were generated from whole embryos and, therefore, the cell type is not known *a priori*, although subsequent analysis of S2 cells suggest they are of macrophage origin [33]. All lines showed doubling times between 31 h and 43 h at 22°C during the steepest part of the growth curve and achieved a broad range of confluent densities (Table 2). Differences in doubling times and confluent densities could reflect in part the different cell types present in the lines, but it is also likely that there is a genetic component conferred by the *Pten* mutation. The *Pten^117^* mutant lines had the longest doubling times (Table 2). The *Pten^117^* and the *Pten^117^*; *Ras^V12^* expressing lines mutant lines also had significantly lower confluent densities than the *Ras^V12^* expressing lines (Table 2). Thus cells with a mutation in *Pten* fail to reach high confluent densities even in the presence of *Ras^V12^*. Further analysis will be required to determine the molecular basis for the effect. By the measures of doubling time and confluent density alone, the *Pten^117^* lines have less practical use because growing the cells takes longer and not as many cells can be generated in a culture vessel. However, as discussed below there are likely to be cases in which having an additional genetic method to generate cell lines will be critical. *Pten^117^* mutant cell cultures will also be key reagents for studying the immortalization process itself.

Western analysis of the lines showed the expected pathways were strongly activated; Akt in *Pten^117^* mutant lines, Erk in the *Ras^V12^* expressing lines and both in the *Pten^117^; Ras^V12^* lines (Figure 3I). S2 cells showed only basal activation of Erk (Figure 3I). The Ras 7 line showed activation of PI3K (Figure 3I), as has been noted for cells expressing the *Ras^V12^* allele *in vivo* [30], [31] and *in vitro* [4]. Cells of the line Pten X, showed higher levels of Erk activation than the other two Pten lines demonstrating that individual lines can differ presumably either due to the specific cell type present in the line or through additional genetic changes that occur during prolonged culture (Figure 3I). In S2 cells there was no activation of Akt, which is a target of insulin signaling, and only weak activity of Erk signaling (Figure 3I). Analysis of S2 cells has shown that the insulin receptor is expressed, but the pathway is unlikely to be active because the ligands are not expressed [34]. The PVR pathway is thought to be active and could be contributing to the activation of Erk we observed [34].

### Concluding remarks

Our major finding is that *Pten* loss promotes cell proliferation *in vitro* and allows the rapid derivation of cell lines, providing another genetic method to generate cell lines in *Drosophila* in addition to the use of oncogenic *Ras^V12^* [4]. We have successfully used expression of transgenic *Ras^V12^* to derive cell lines from the *rumi* mutant [8]. The cells, which are null for *rumi* have been used to study Notch signaling *in vitro* [9]. In a similar fashion *Pten* mutations could be used to establish cell lines corresponding to mutants. This is important because it provides an alternate signaling context in which to study a mutant where having the Ras pathway active would interfere with the analysis.

## Materials and Methods

### Fly stocks

*CyO, Act5C-GFP*; *TM6B, Ubi-GFP*; *FM7i, Act-GFP*, *crb^2^* and *l(2)gl^4^* flies were obtained from the Bloomington *Drosophila* Stock Center. The GFP-marked balancer chromosomes were used to generate stocks of the tumor suppressor mutations. The following alleles were obtained from colleagues: *hpo^BF33^*, J. Jiang [35]; *wts^MGH1^*, D.J. Pan [36]; *brat^11^*, D. Frank [37]; *Pten^117^*, H. Stocker [29]; *hyd^35^*, J. Treisman [20]; *scrib* ^1^, D. Bilder [38]; *dlg^XI-2^*, V. Budnik [39]. *Pten^117^*, UAS-*RAS^V12^* and *Pten^117^*, *Act5C-Gal4* recombinant chromosomes were generated using standard crosses.

### Cell culture

Primary cultures were established using a standard method [4]. Schneider's medium with 10% heat inactivated Fetal Bovine serum was used for all cell culture work. Multiple primary cultures were generated for each genotype and examined to document the development of various cells types. The time at which patches of dividing cells emerged was determined. Cultures of *Pten* ^117^ and *Pten* ^117^; *Ras^V12^* cells were maintained until they reached confluence and were sub-cultured to generate cell lines using standard methods [4].

### Cell line characterization

For growth curves, cells were plated at 2.5×10^5^ cells/well in a 12-well plate with 1 ml of medium. Duplicate cell counts were made over 7 days. Doubling time was determined during the period when cells numbers increased most steeply in a given line (typically days 3–5). Confluent densities were determined at the point when there was no further increase in cell number.

### Genotyping of Pten mutant cell lines

The *Pten^117^* allele has a deletion of 5 base pairs in the coding region of the gene that results in a frame-shift and insertion of a premature stop codon. DNA from *Pten* cultures was amplified using the primers shown below. The amplified DNA was purified and sequenced to confirm presence of the mutation.

5′-GCGAAAGTTCATAAATATCATGGC, 3′-CGGCGCTGAATGTGGCGC.

### Western Analysis

Cells were lysed in TN1 buffer containing 125 mM NaCl, 50 mM Tris (pH = 8.0), 10 mM EDTA (pH = 8.0), 10 mM Na_4_P_2_O_7_ ·10H_2_O, 10 mM NaF, 1% Triton X-100, 3 mM Na_3_VO_4_ supplemented with protease inhibitor cocktail (Roche Diagnostics Corp. Indianapolis, IN), centrifuged, and supernatants were used for analysis. Total protein (10 µg) was separated on polyacrylamide gels and immunoblots were incubated with antibodies directed against *Drosophila*-specific Akt and pAkt (Ser 505) (Cell Signaling Technology; Danvers, MA), pan-Erk, (Santa Cruz Biotechnology; Santa Cruz, CA) and dpErk1/2 (E10) (Cell Signaling Technology; Danvers, MA).

## Supporting Information

Figure S1 **Statistical analysis of cell proliferation data.** Box and whisker plot of the data from Figure 1B in the main text showing the time for proliferating cells to appear in primary cultures of different genotypes. Boxes are drawn between the mean and the median. The whiskers end at the maximum and minimum values in the sample population. Samples were analyzed using Dunn's multiple comparison test post the Kruskal-Wallis test. Pairs that were identified as significantly different are connected by solid lines. Both *Pten* mutant and *Ras^V12^* expressing cultures are significantly different than wild-type cultures (P<0.001). Both *hpo* and *wts* cultures are significantly different than wild-type cultures (P<0.05).
(PDF)
