# Supplementary material for: Loss of the Tumor Suppressor Pten Promotes Proliferation of Drosophila melanogaster Cells In Vitro and Gives Rise to Continuous Cell Lines
Source: PLoS One. 2012 Feb 21;7(2):e31417. doi: 10.1371/journal.pone.0031417 (PMC3283623; doi:10.1371/journal.pone.0031417)
Supplement: Figure S1 — Statistical analysis of cell proliferation data. Box and whisker plot of the data from Figure 1B in the main text showing the time for proliferating cells to appear in primary cultures of different genotypes. Boxes are drawn between the mean and the median. The whiskers end at the maximum and minimum values in the sample population. Samples were analyzed using Dunn's multiple comparison test post the Kruskal-Wallis test. Pairs that were identified as significantly different are connected by solid lines. Both Pten mutant and RasV12 expressing cultures are significantly different than wild-type cultures (P<0.001). Both hpo and wts cultures are significantly different than wild-type cultures (P<0.05). (PDF) [file pone.0031417.s001.pdf]

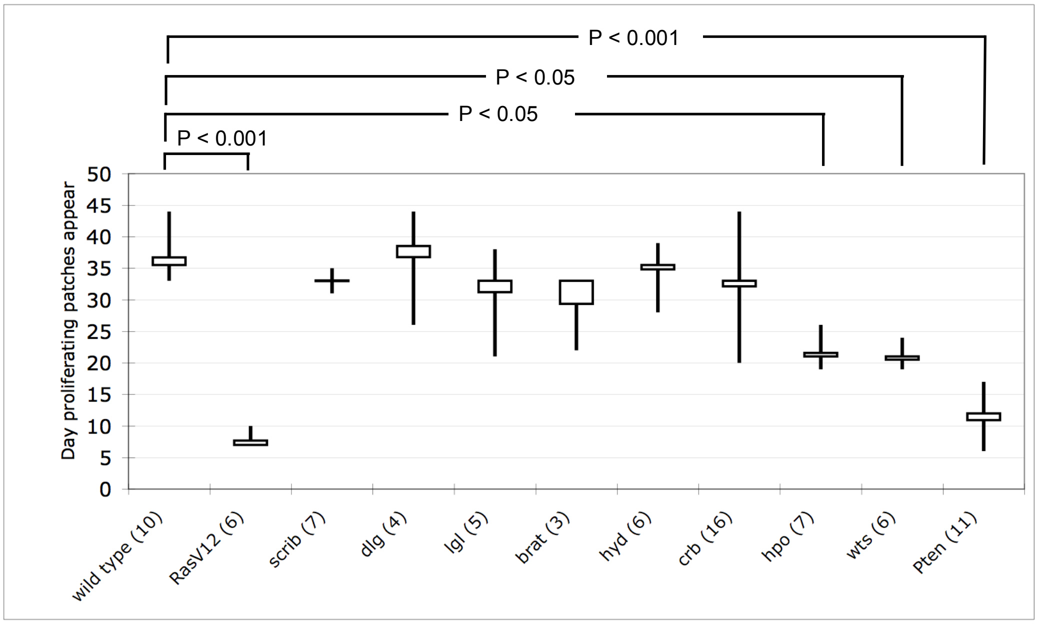


**Supplementary Figure S1. Statistical analysis of cell proliferation data.** Box and whisker plot of the data from Figure 1B in the main text showing the time for proliferating cells to appear in primary cultures of different genotypes. Boxes are drawn between the mean and the median. The whiskers end at the maximum and minimum values in the sample population. Samples were analyzed using Dunn's multiple comparison test post the Kruskal-Wallis test. Pairs that were identified as significantly different are connected by solid lines. Both *Pten* mutant and $Ras^{V12}$ expressing cultures are significantly different than wild-type cultures ($P < 0.001$). Both *hpo* and *wts* cultures are significantly different than wild-type cultures ($P < 0.05$).
